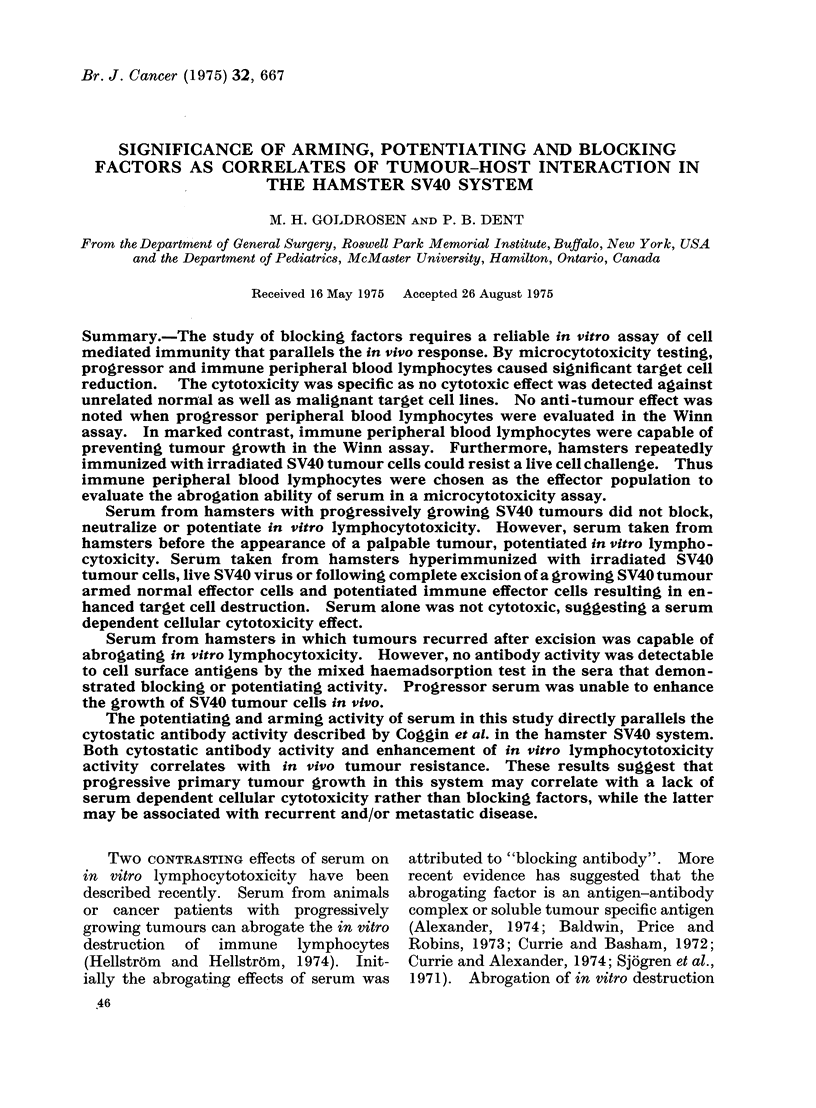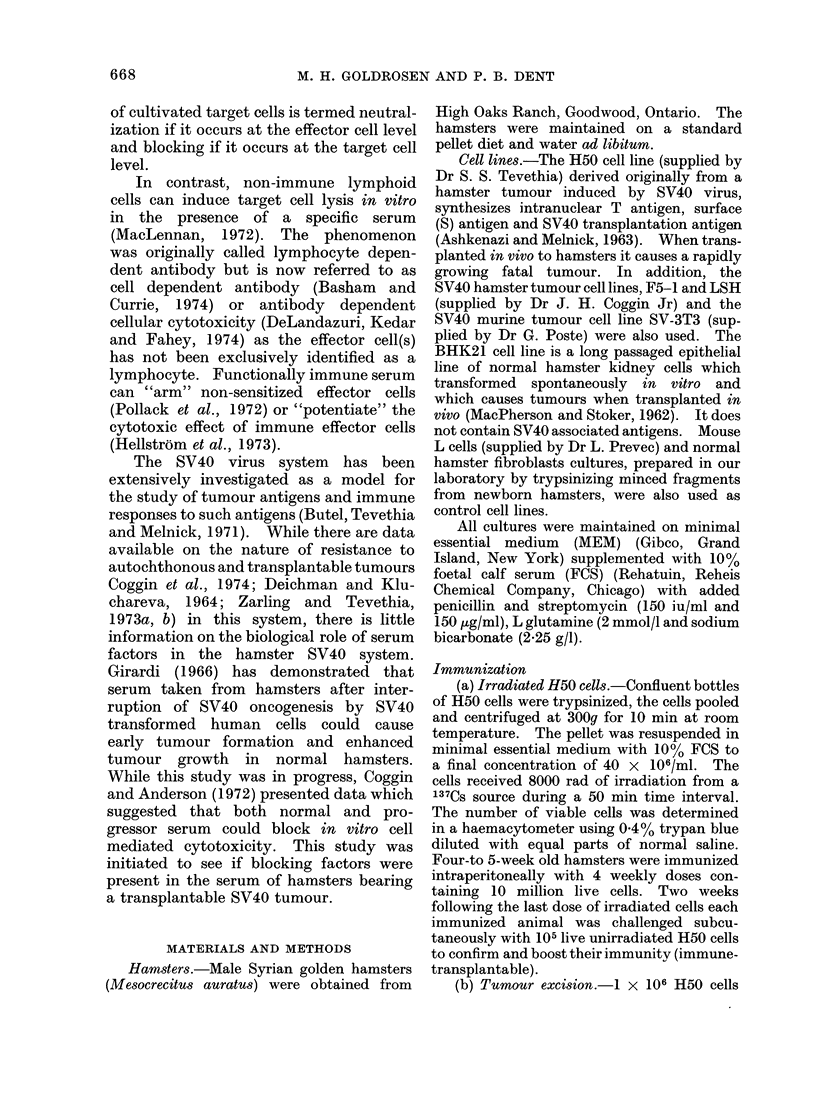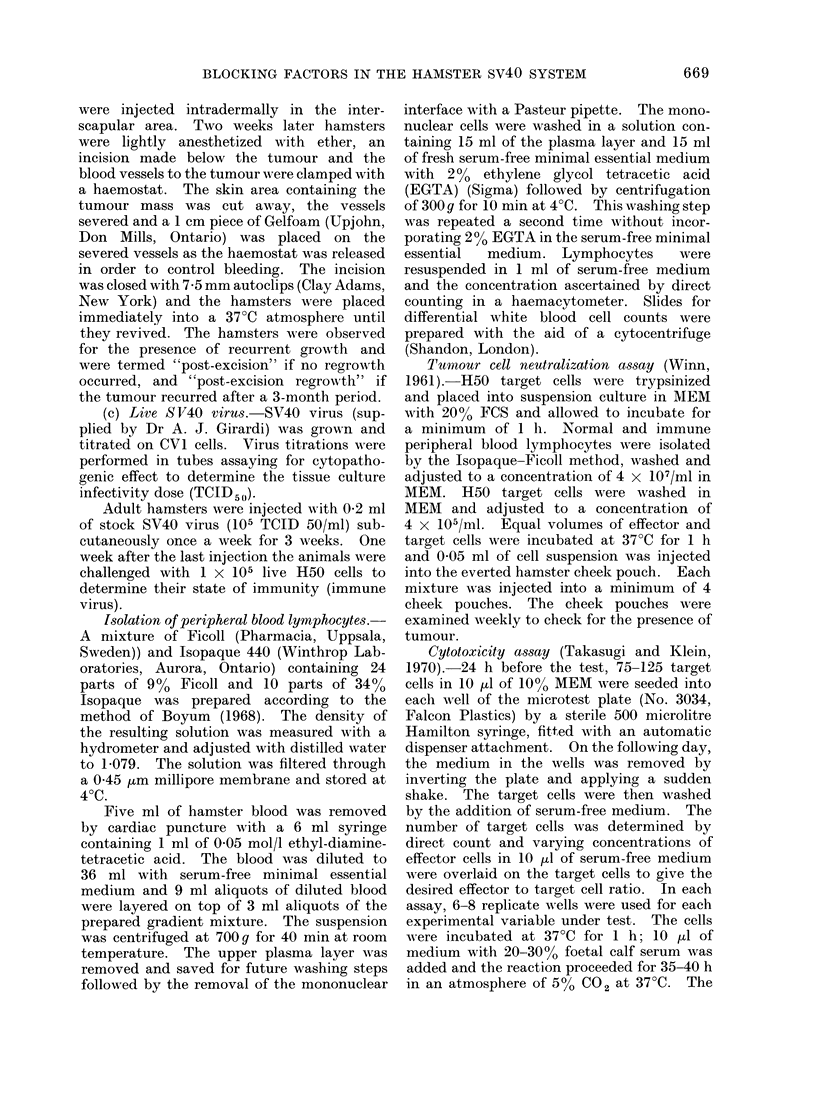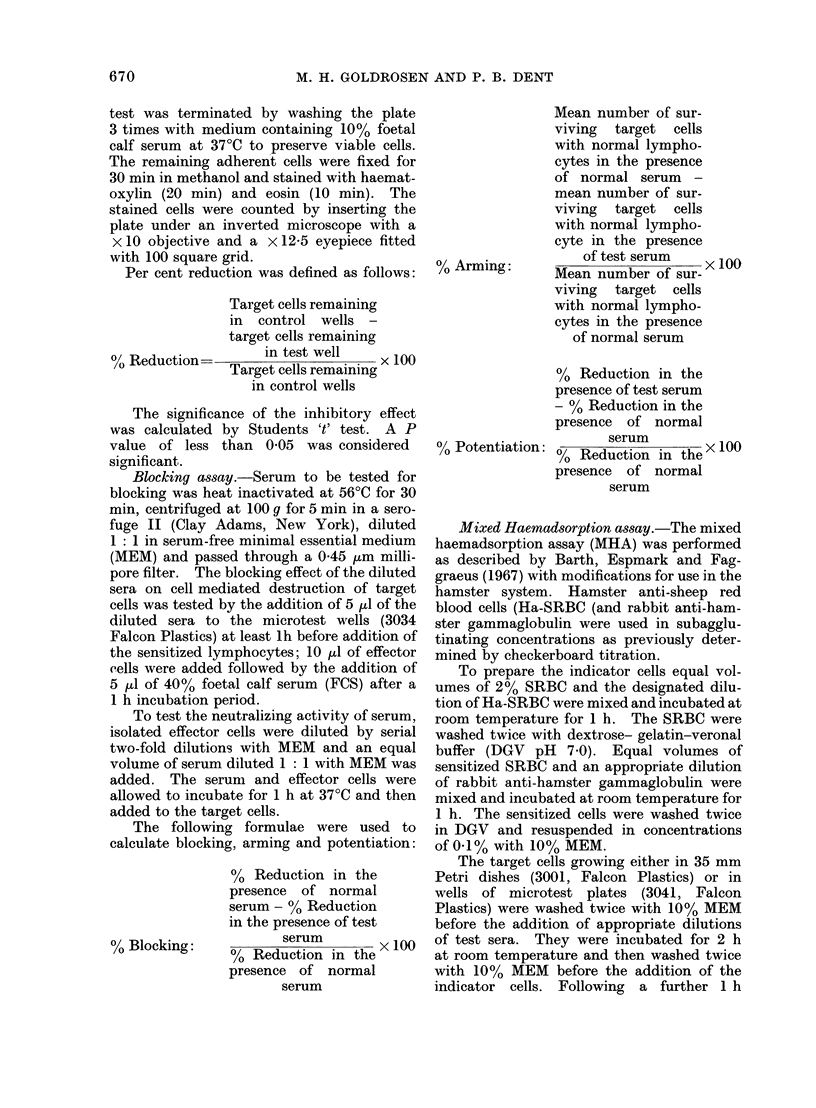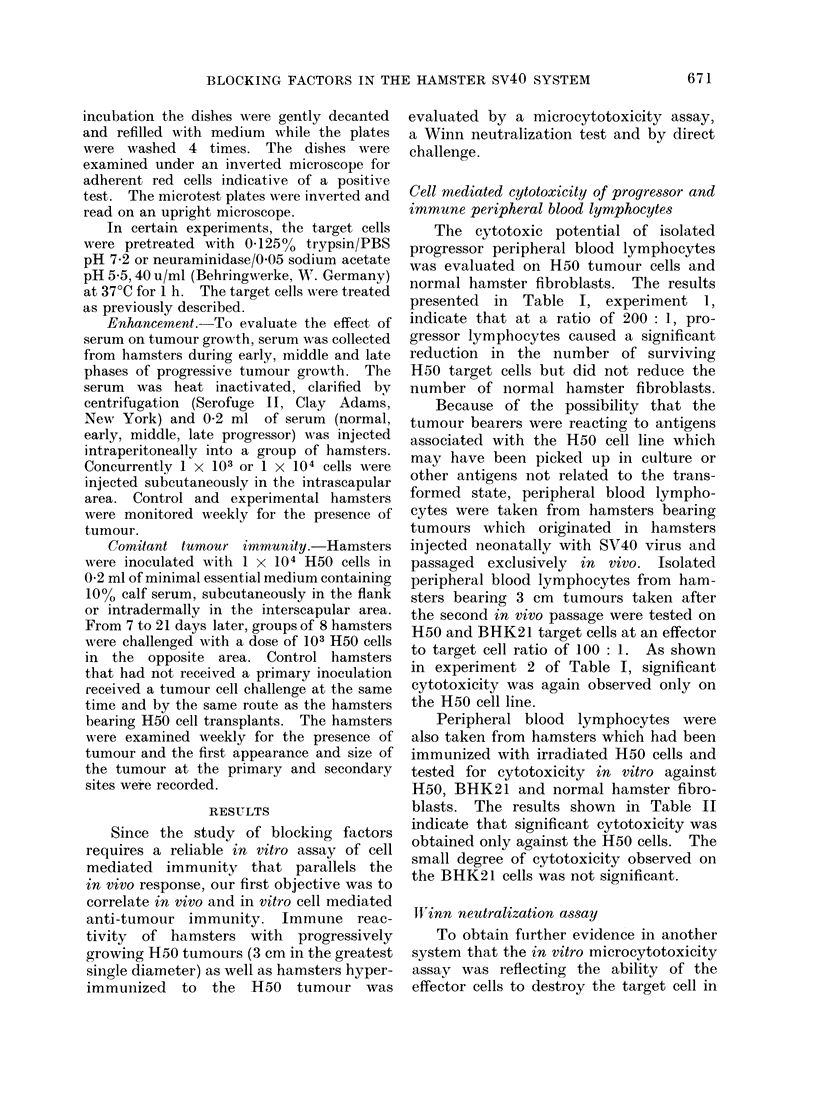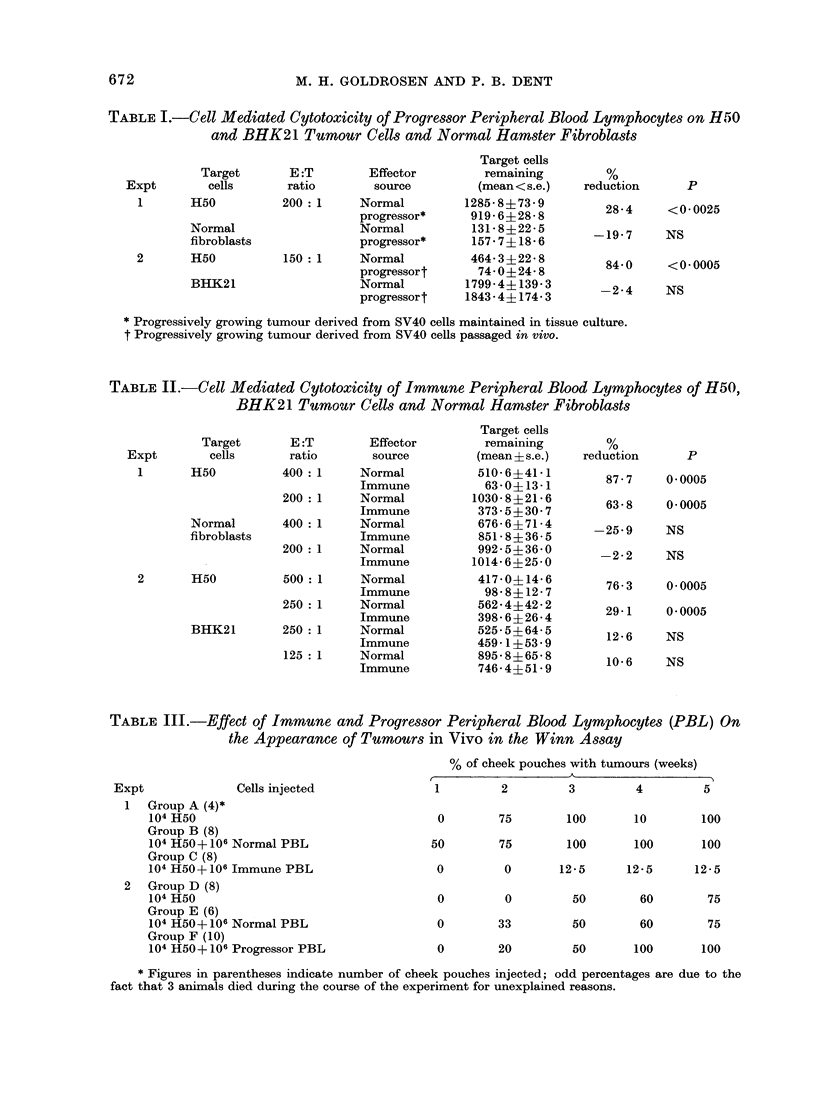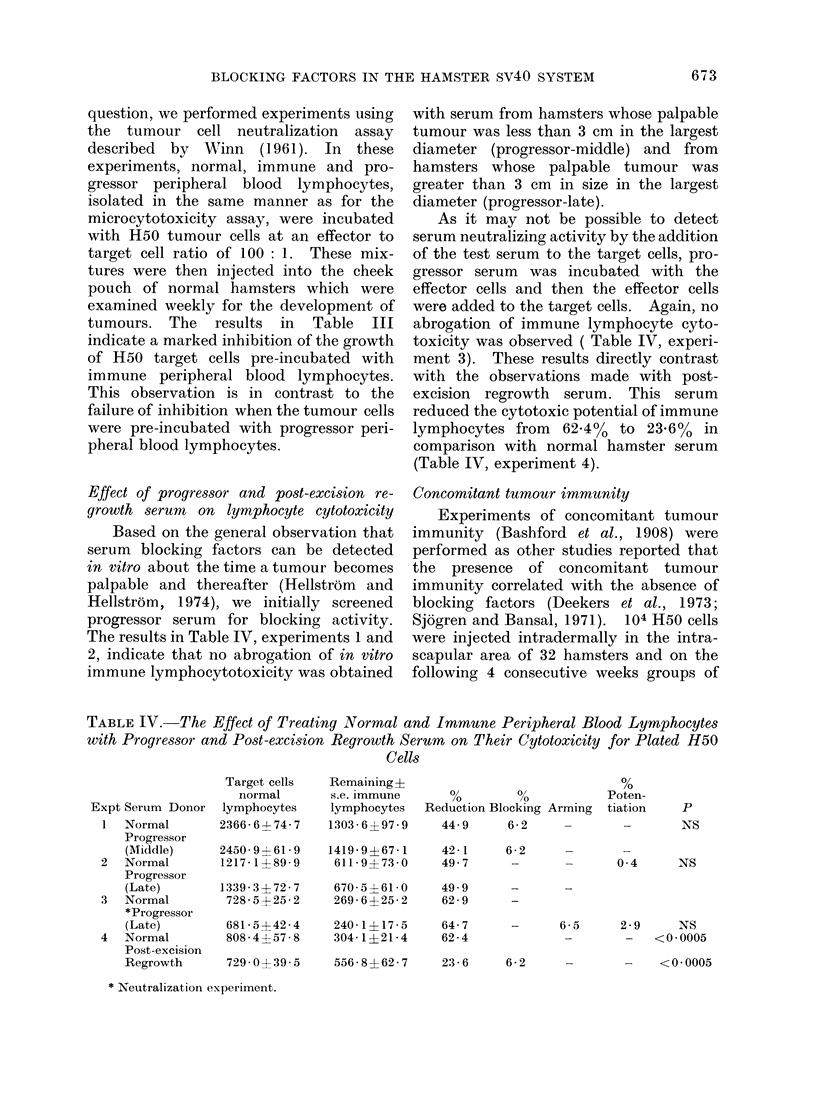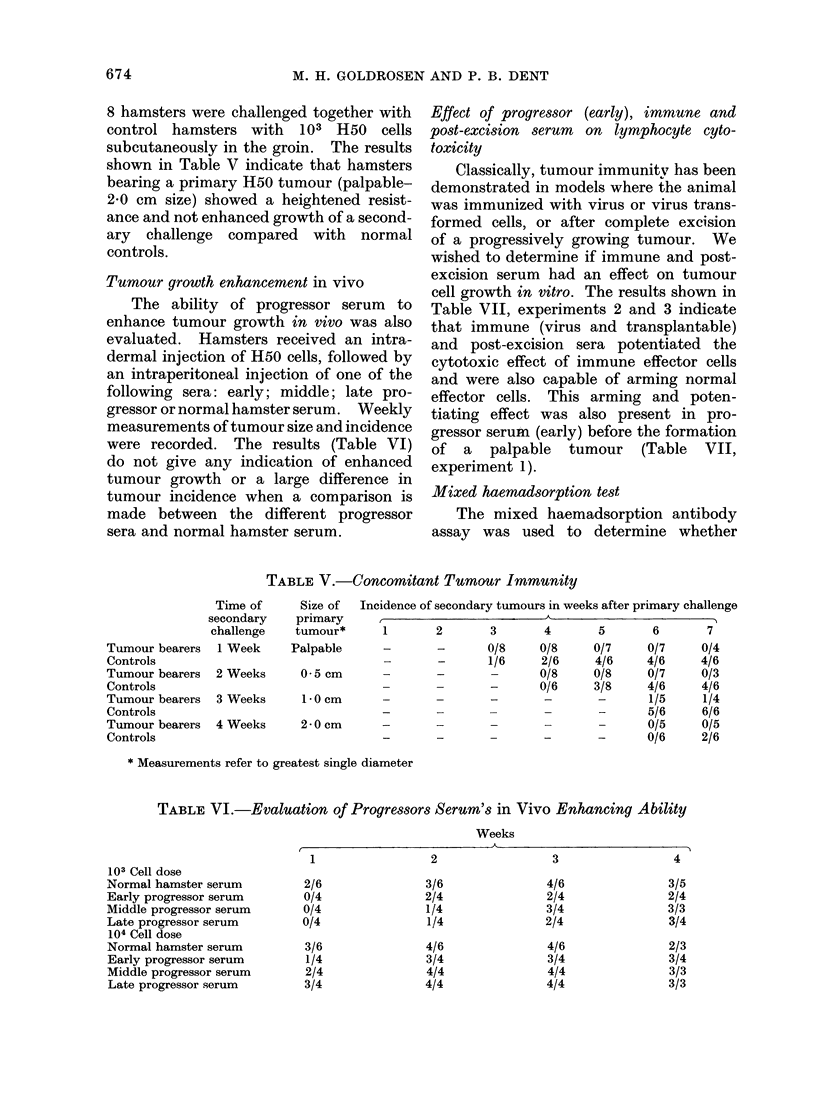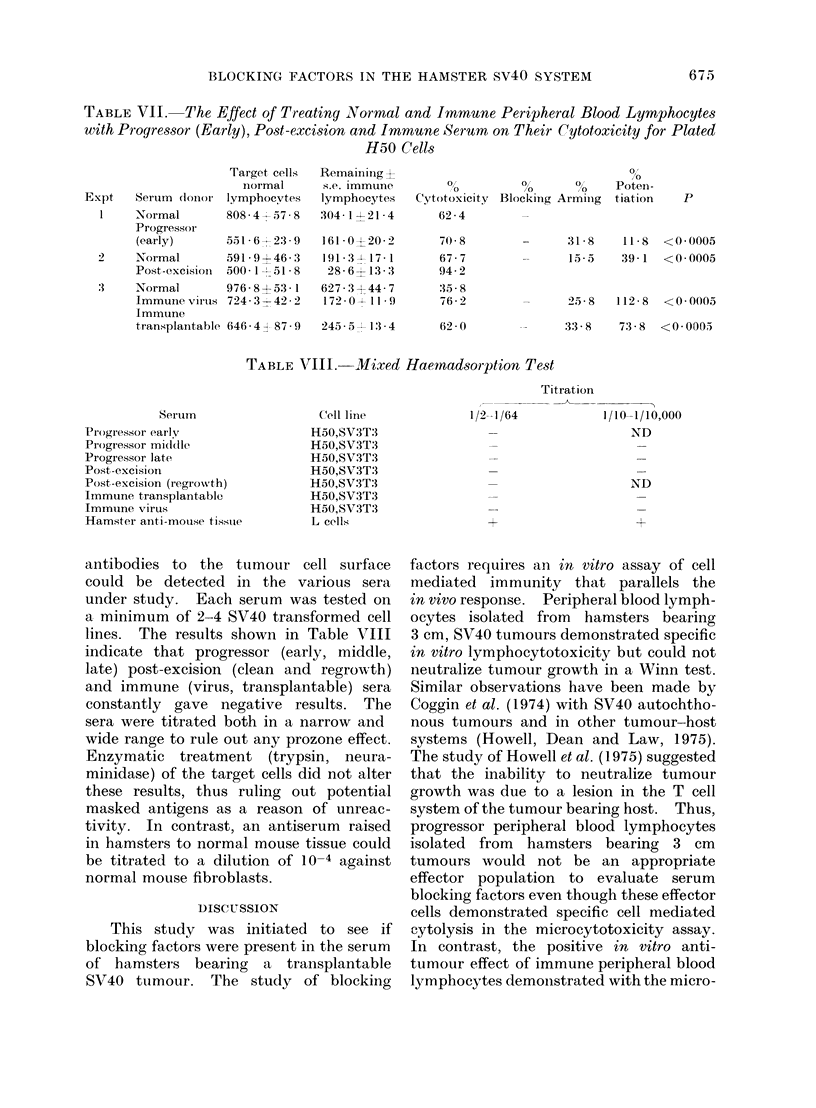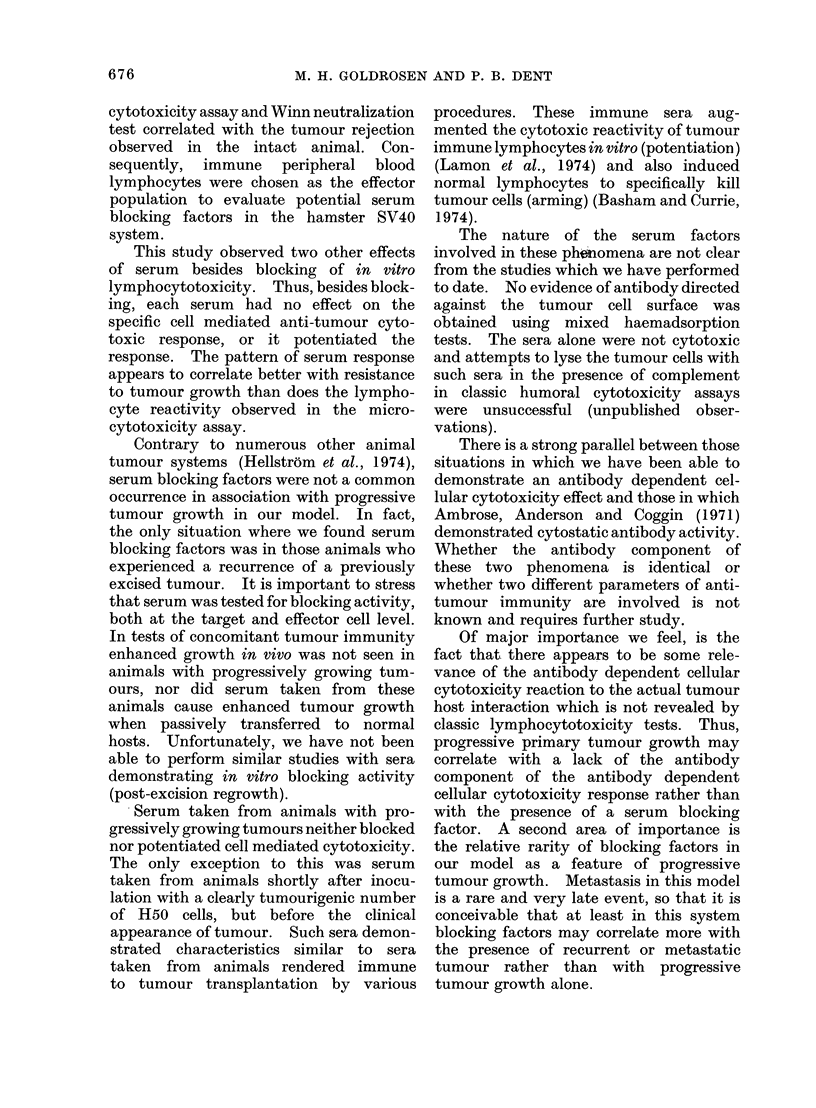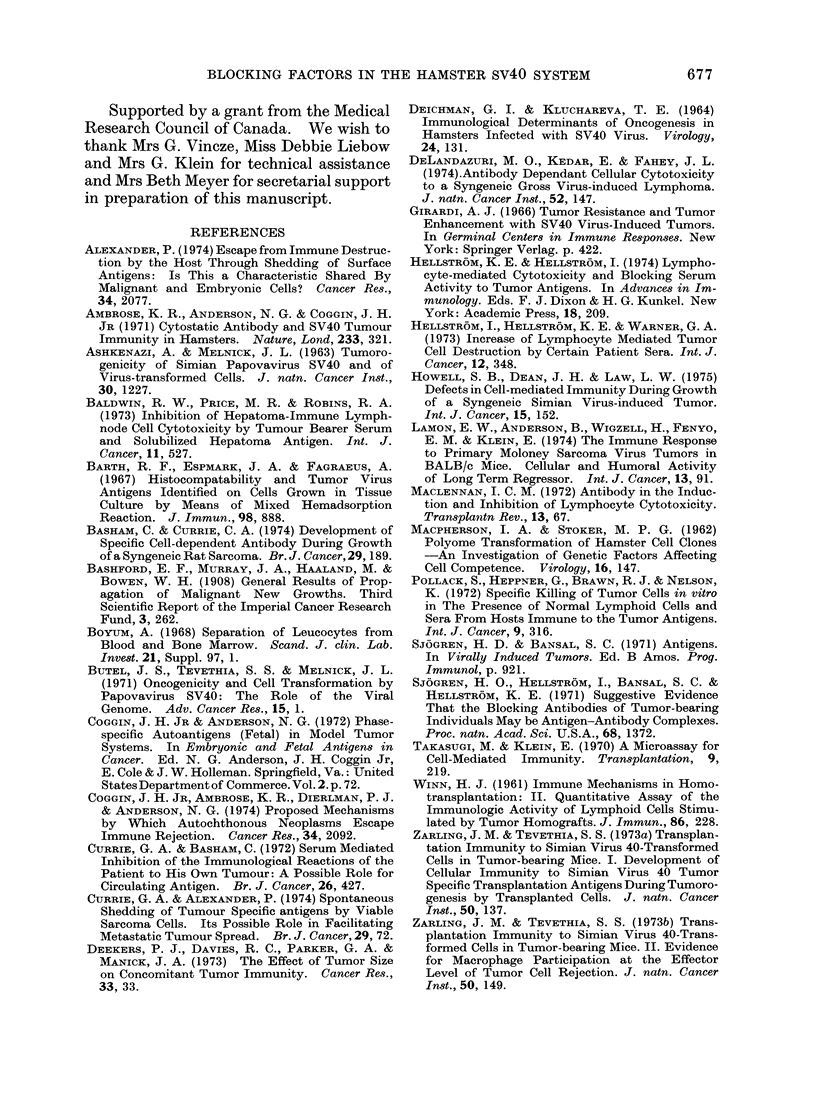# Significance of arming, potentiating and blocking factors as correlates the tumour-host interaction in the hamster SV40 system.

**DOI:** 10.1038/bjc.1975.277

**Published:** 1975-12

**Authors:** M. H. Goldrosen, P. B. Dent

## Abstract

The study of blocking factors requires in vitro assay of cell mediated immunity that parallels the in vivo response. By microcytotoxicity testing, progressor and immune peripheral blood lymphocytes caused significant target cell reduction. The cytotoxicity was specific as no cytotoxic effect was detected against unrelated normal as well as a malignant target cell lines. No anti-tumour effect was noted when progressor peripheral blood lymphocytes were evaluated in the Winn assay. In marked contrast, immune peripheral blood lymphocytes were capable of preventing tumour growth in the Winn assay. Furthermore, hamsters repeatedly immunized with irradiated SV40 tumour cells could resist a live cell challenge. Thus immune peripheral blood lymphocytes were chosen as the effector population to evaluate the abrogation ability of serum in the microcytotoxicity assay.


*Br. J. Cancer* (1975) **32**, 667

### SIGNIFICANCE OF ARMING, POTENTIATING AND BLOCKING FACTORS AS CORRELATES OF TUMOUR–HOST INTERACTION IN THE HAMSTER SV40 SYSTEM

M. H. GOLDROSEN AND P. B. DENT

*From the Department of General Surgery, Roswell Park Memorial Institute, Buffalo, New York, USA and the Department of Pediatrics, McMaster University, Hamilton, Ontario, Canada*

Received 16 May 1975 Accepted 26 August 1975

**Summary.—The study of blocking factors requires a reliable *in vitro* assay of cell mediated immunity that parallels the *in vivo* response. By microcytotoxicity testing, progressor and immune peripheral blood lymphocytes caused significant target cell reduction. The cytotoxicity was specific as no cytotoxic effect was detected against unrelated normal as well as malignant target cell lines. No anti-tumour effect was noted when progressor peripheral blood lymphocytes were evaluated in the Winn assay. In marked contrast, immune peripheral blood lymphocytes were capable of preventing tumour growth in the Winn assay. Furthermore, hamsters repeatedly immunized with irradiated SV40 tumour cells could resist a live cell challenge. Thus immune peripheral blood lymphocytes were chosen as the effector population to evaluate the abrogation ability of serum in a microcytotoxicity assay.**

**Serum from hamsters with progressively growing SV40 tumours did not block, neutralize or potentiate *in vitro* lymphocytotoxicity. However, serum taken from hamsters before the appearance of a palpable tumour, potentiated *in vitro* lymphocytoxicity. Serum taken from hamsters hyperimmunized with irradiated SV40 tumour cells, live SV40 virus or following complete excision of a growing SV40 tumour armed normal effector cells and potentiated immune effector cells resulting in enhanced target cell destruction. Serum alone was not cytotoxic, suggesting a serum dependent cellular cytotoxicity effect.**

**Serum from hamsters in which tumours recurred after excision was capable of abrogating *in vitro* lymphocytoxicity. However, no antibody activity was detectable to cell surface antigens by the mixed haemadsorption test in the sera that demonstrated blocking or potentiating activity. Progressor serum was unable to enhance the growth of SV40 tumour cells *in vivo*.**

**The potentiating and arming activity of serum in this study directly parallels the cytostatic antibody activity described by Coggin *et al.* in the hamster SV40 system. Both cytostatic antibody activity and enhancement of *in vitro* lymphocytotoxicity activity correlates with *in vivo* tumour resistance. These results suggest that progressive primary tumour growth in this system may correlate with a lack of serum dependent cellular cytotoxicity rather than blocking factors, while the latter may be associated with recurrent and/or metastatic disease.**

TWO CONTRASTING effects of serum on *in vitro* lymphocytotoxicity have been described recently. Serum from animals or cancer patients with progressively growing tumours can abrogate the *in vitro* destruction of immune lymphocytes (Hellström and Hellström, 1974). Initially the abrogating effects of serum was attributed to "blocking antibody". More recent evidence has suggested that the abrogating factor is an antigen–antibody complex or soluble tumour specific antigen (Alexander, 1974; Baldwin, Price and Robins, 1973; Currie and Basham, 1972; Currie and Alexander, 1974; Sjögren *et al.*, 1971). Abrogation of *in vitro* destruction of cultivated target cells is termed neutralization if it occurs at the effector cell level and blocking if it occurs at the target cell level.

In contrast, non-immune lymphoid cells can induce target cell lysis *in vitro* in the presence of a specific serum (MacLennan, 1972). The phenomenon was originally called lymphocyte dependent antibody but is now referred to as cell dependent antibody (Basham and Currie, 1974) or antibody dependent cellular cytotoxicity (DeLandazuri, Kedar and Fahey, 1974) as the effector cell(s) has not been exclusively identified as a lymphocyte. Functionally immune serum can "arm" non-sensitized effector cells (Pollack *et al.*, 1972) or "potentiate" the cytotoxic effect of immune effector cells (Hellström *et al.*, 1973).

The SV40 virus system has been extensively investigated as a model for the study of tumour antigens and immune responses to such antigens (Butel, Tevethia and Melnick, 1971). While there are data available on the nature of resistance to autochthonous and transplantable tumours Coggin *et al.*, 1974; Deichman and Kluchareva, 1964; Zarling and Tevethia, 1973*a*, *b*) in this system, there is little information on the biological role of serum factors in the hamster SV40 system. Girardi (1966) has demonstrated that serum taken from hamsters after interruption of SV40 oncogenesis by SV40 transformed human cells could cause early tumour formation and enhanced tumour growth in normal hamsters. While this study was in progress, Coggin and Anderson (1972) presented data which suggested that both normal and progressor serum could block *in vitro* cell mediated cytotoxicity. This study was initiated to see if blocking factors were present in the serum of hamsters bearing a transplantable SV40 tumour.

### MATERIALS AND METHODS

*Hamsters.*—Male Syrian golden hamsters (*Mesocrecitus auratus*) were obtained from High Oaks Ranch, Goodwood, Ontario. The hamsters were maintained on a standard pellet diet and water *ad libitum.*

*Cell lines.*—The H50 cell line (supplied by Dr S. S. Tevethia) derived originally from a hamster tumour induced by SV40 virus, synthesizes intranuclear T antigen, surface (S) antigen and SV40 transplantation antigen (Ashkenazi and Melnick, 1963). When transplanted *in vivo* to hamsters it causes a rapidly growing fatal tumour. In addition, the SV40 hamster tumour cell lines, F5-1 and LSH (supplied by Dr J. H. Coggin Jr) and the SV40 murine tumour cell line SV-3T3 (supplied by Dr G. Poste) were also used. The BHK21 cell line is a long passaged epithelial line of normal hamster kidney cells which transformed spontaneously *in vitro* and which causes tumours when transplanted *in vivo* (MacPherson and Stoker, 1962). It does not contain SV40 associated antigens. Mouse L cells (supplied by Dr L. Prevec) and normal hamster fibroblasts cultures, prepared in our laboratory by trypsinizing minced fragments from newborn hamsters, were also used as control cell lines.

All cultures were maintained on minimal essential medium (MEM) (Gibco, Grand Island, New York) supplemented with 10% foetal calf serum (FCS) (Rehatuin, Reheis Chemical Company, Chicago) with added penicillin and streptomycin (150 iu/ml and 150 $\mu$g/ml), L glutamine (2 mmol/l and sodium bicarbonate (2·25 g/l).

### *Immunization*

(a) *Irradiated H50 cells.*—Confluent bottles of H50 cells were trypsinized, the cells pooled and centrifuged at 300*g* for 10 min at room temperature. The pellet was resuspended in minimal essential medium with 10% FCS to a final concentration of $40 \times 10^6$/ml. The cells received 8000 rad of irradiation from a $^{137}$Cs source during a 50 min time interval. The number of viable cells was determined in a haemacytometer using 0·4% trypan blue diluted with equal parts of normal saline. Four-to 5-week old hamsters were immunized intraperitoneally with 4 weekly doses containing 10 million live cells. Two weeks following the last dose of irradiated cells each immunized animal was challenged subcutaneously with $10^5$ live unirradiated H50 cells to confirm and boost their immunity (immune-transplantable).

(b) *Tumour excision.*—$1 \times 10^6$ H50 cells were injected intradermally in the interscapular area. Two weeks later hamsters were lightly anesthetized with ether, an incision made below the tumour and the blood vessels to the tumour were clamped with a haemostat. The skin area containing the tumour mass was cut away, the vessels severed and a 1 cm piece of Gelfoam (Upjohn, Don Mills, Ontario) was placed on the severed vessels as the haemostat was released in order to control bleeding. The incision was closed with 7·5 mm autoclips (Clay Adams, New York) and the hamsters were placed immediately into a 37°C atmosphere until they revived. The hamsters were observed for the presence of recurrent growth and were termed "post-excision" if no regrowth occurred, and "post-excision regrowth" if the tumour recurred after a 3-month period.

(c) *Live SV40 virus.*—SV40 virus (supplied by Dr A. J. Girardi) was grown and titrated on CV1 cells. Virus titrations were performed in tubes assaying for cytopathogenic effect to determine the tissue culture infectivity dose ($TCID_{50}$).

Adult hamsters were injected with 0·2 ml of stock SV40 virus ($10^5$ TCID 50/ml) subcutaneously once a week for 3 weeks. One week after the last injection the animals were challenged with $1 \times 10^5$ live H50 cells to determine their state of immunity (immune virus).

*Isolation of peripheral blood lymphocytes.*—A mixture of Ficoll (Pharmacia, Uppsala, Sweden)) and Isopaque 440 (Winthrop Laboratories, Aurora, Ontario) containing 24 parts of 9% Ficoll and 10 parts of 34% Isopaque was prepared according to the method of Boyum (1968). The density of the resulting solution was measured with a hydrometer and adjusted with distilled water to 1·079. The solution was filtered through a 0·45 $\mu$m millipore membrane and stored at 4°C.

Five ml of hamster blood was removed by cardiac puncture with a 6 ml syringe containing 1 ml of 0·05 mol/l ethyl-diamine-tetracetic acid. The blood was diluted to 36 ml with serum-free minimal essential medium and 9 ml aliquots of diluted blood were layered on top of 3 ml aliquots of the prepared gradient mixture. The suspension was centrifuged at 700 *g* for 40 min at room temperature. The upper plasma layer was removed and saved for future washing steps followed by the removal of the mononuclear interface with a Pasteur pipette. The mononuclear cells were washed in a solution containing 15 ml of the plasma layer and 15 ml of fresh serum-free minimal essential medium with 2% ethylene glycol tetracetic acid (EGTA) (Sigma) followed by centrifugation of 300 *g* for 10 min at 4°C. This washing step was repeated a second time without incorporating 2% EGTA in the serum-free minimal essential medium. Lymphocytes were resuspended in 1 ml of serum-free medium and the concentration ascertained by direct counting in a haemacytometer. Slides for differential white blood cell counts were prepared with the aid of a cytocentrifuge (Shandon, London).

*Tumour cell neutralization assay* (Winn, 1961).—H50 target cells were trypsinized and placed into suspension culture in MEM with 20% FCS and allowed to incubate for a minimum of 1 h. Normal and immune peripheral blood lymphocytes were isolated by the Isopaque–Ficoll method, washed and adjusted to a concentration of $4 \times 10^7$/ml in MEM. H50 target cells were washed in MEM and adjusted to a concentration of $4 \times 10^5$/ml. Equal volumes of effector and target cells were incubated at 37°C for 1 h and 0·05 ml of cell suspension was injected into the everted hamster cheek pouch. Each mixture was injected into a minimum of 4 cheek pouches. The cheek pouches were examined weekly to check for the presence of tumour.

*Cytotoxicity assay* (Takasugi and Klein, 1970).—24 h before the test, 75–125 target cells in 10 $\mu$l of 10% MEM were seeded into each well of the microtest plate (No. 3034, Falcon Plastics) by a sterile 500 microlitre Hamilton syringe, fitted with an automatic dispenser attachment. On the following day, the medium in the wells was removed by inverting the plate and applying a sudden shake. The target cells were then washed by the addition of serum-free medium. The number of target cells was determined by direct count and varying concentrations of effector cells in 10 $\mu$l of serum-free medium were overlaid on the target cells to give the desired effector to target cell ratio. In each assay, 6–8 replicate wells were used for each experimental variable under test. The cells were incubated at 37°C for 1 h; 10 $\mu$l of medium with 20–30% foetal calf serum was added and the reaction proceeded for 35–40 h in an atmosphere of 5% $CO_2$ at 37°C. The test was terminated by washing the plate 3 times with medium containing 10% foetal calf serum at 37°C to preserve viable cells. The remaining adherent cells were fixed for 30 min in methanol and stained with haematoxylin (20 min) and eosin (10 min). The stained cells were counted by inserting the plate under an inverted microscope with a ×10 objective and a ×12·5 eyepiece fitted with 100 square grid.

Per cent reduction was defined as follows:

$$\% \text{ Reduction} = \frac{\text{Target cells remaining in control wells} - \text{target cells remaining in test well}}{\text{Target cells remaining in control wells}} \times 100$$

The significance of the inhibitory effect was calculated by Students '*t*' test. A *P* value of less than 0·05 was considered significant.

*Blocking assay.*—Serum to be tested for blocking was heat inactivated at 56°C for 30 min, centrifuged at 100 *g* for 5 min in a serofuge II (Clay Adams, New York), diluted 1 : 1 in serum-free minimal essential medium (MEM) and passed through a 0·45 μm millipore filter. The blocking effect of the diluted sera on cell mediated destruction of target cells was tested by the addition of 5 μl of the diluted sera to the microtest wells (3034 Falcon Plastics) at least 1h before addition of the sensitized lymphocytes; 10 μl of effector cells were added followed by the addition of 5 μl of 40% foetal calf serum (FCS) after a 1 h incubation period.

To test the neutralizing activity of serum, isolated effector cells were diluted by serial two-fold dilutions with MEM and an equal volume of serum diluted 1 : 1 with MEM was added. The serum and effector cells were allowed to incubate for 1 h at 37°C and then added to the target cells.

The following formulae were used to calculate blocking, arming and potentiation:

$$\% \text{ Blocking:} \quad \frac{\% \text{ Reduction in the presence of normal serum} - \% \text{ Reduction in the presence of test serum}}{\% \text{ Reduction in the presence of normal serum}} \times 100$$

$$\% \text{ Arming:} \quad \frac{\text{Mean number of surviving target cells with normal lymphocytes in the presence of normal serum} - \text{mean number of surviving target cells with normal lymphocyte in the presence of test serum}}{\text{Mean number of surviving target cells with normal lymphocytes in the presence of normal serum}} \times 100$$

$$\% \text{ Potentiation:} \quad \frac{\% \text{ Reduction in the presence of test serum} - \% \text{ Reduction in the presence of normal serum}}{\% \text{ Reduction in the presence of normal serum}} \times 100$$

*Mixed Haemadsorption assay.*—The mixed haemadsorption assay (MHA) was performed as described by Barth, Espmark and Faggraeus (1967) with modifications for use in the hamster system. Hamster anti-sheep red blood cells (Ha-SRBC (and rabbit anti-hamster gammaglobulin were used in subagglutinating concentrations as previously determined by checkerboard titration.

To prepare the indicator cells equal volumes of 2% SRBC and the designated dilution of Ha-SRBC were mixed and incubated at room temperature for 1 h. The SRBC were washed twice with dextrose– gelatin–veronal buffer (DGV pH 7·0). Equal volumes of sensitized SRBC and an appropriate dilution of rabbit anti-hamster gammaglobulin were mixed and incubated at room temperature for 1 h. The sensitized cells were washed twice in DGV and resuspended in concentrations of 0·1% with 10% MEM.

The target cells growing either in 35 mm Petri dishes (3001, Falcon Plastics) or in wells of microtest plates (3041, Falcon Plastics) were washed twice with 10% MEM before the addition of appropriate dilutions of test sera. They were incubated for 2 h at room temperature and then washed twice with 10% MEM before the addition of the indicator cells. Following a further 1 h incubation the dishes were gently decanted and refilled with medium while the plates were washed 4 times. The dishes were examined under an inverted microscope for adherent red cells indicative of a positive test. The microtest plates were inverted and read on an upright microscope.

In certain experiments, the target cells were pretreated with 0·125% trypsin/PBS pH 7·2 or neuraminidase/0·05 sodium acetate pH 5·5, 40 u/ml (Behringwerke, W. Germany) at 37°C for 1 h. The target cells were treated as previously described.

*Enhancement.*—To evaluate the effect of serum on tumour growth, serum was collected from hamsters during early, middle and late phases of progressive tumour growth. The serum was heat inactivated, clarified by centrifugation (Serofuge II, Clay Adams, New York) and 0·2 ml of serum (normal, early, middle, late progressor) was injected intraperitoneally into a group of hamsters. Concurrently $1 \times 10^3$ or $1 \times 10^4$ cells were injected subcutaneously in the intrascapular area. Control and experimental hamsters were monitored weekly for the presence of tumour.

*Comitant tumour immunity.*—Hamsters were inoculated with $1 \times 10^4$ H50 cells in 0·2 ml of minimal essential medium containing 10% calf serum, subcutaneously in the flank or intradermally in the interscapular area. From 7 to 21 days later, groups of 8 hamsters were challenged with a dose of $10^3$ H50 cells in the opposite area. Control hamsters that had not received a primary inoculation received a tumour cell challenge at the same time and by the same route as the hamsters bearing H50 cell transplants. The hamsters were examined weekly for the presence of tumour and the first appearance and size of the tumour at the primary and secondary sites were recorded.

### RESULTS

Since the study of blocking factors requires a reliable *in vitro* assay of cell mediated immunity that parallels the *in vivo* response, our first objective was to correlate *in vivo* and in *vitro* cell mediated anti-tumour immunity. Immune reactivity of hamsters with progressively growing H50 tumours (3 cm in the greatest single diameter) as well as hamsters hyperimmunized to the H50 tumour was evaluated by a microcytotoxicity assay, a Winn neutralization test and by direct challenge.

### *Cell mediated cytotoxicity of progressor and immune peripheral blood lymphocytes*

The cytotoxic potential of isolated progressor peripheral blood lymphocytes was evaluated on H50 tumour cells and normal hamster fibroblasts. The results presented in Table I, experiment 1, indicate that at a ratio of 200 : 1, progressor lymphocytes caused a significant reduction in the number of surviving H50 target cells but did not reduce the number of normal hamster fibroblasts.

Because of the possibility that the tumour bearers were reacting to antigens associated with the H50 cell line which may have been picked up in culture or other antigens not related to the transformed state, peripheral blood lymphocytes were taken from hamsters bearing tumours which originated in hamsters injected neonatally with SV40 virus and passaged exclusively *in vivo*. Isolated peripheral blood lymphocytes from hamsters bearing 3 cm tumours taken after the second *in vivo* passage were tested on H50 and BHK21 target cells at an effector to target cell ratio of 100 : 1. As shown in experiment 2 of Table I, significant cytotoxicity was again observed only on the H50 cell line.

Peripheral blood lymphocytes were also taken from hamsters which had been immunized with irradiated H50 cells and tested for cytotoxicity *in vitro* against H50, BHK21 and normal hamster fibroblasts. The results shown in Table II indicate that significant cytotoxicity was obtained only against the H50 cells. The small degree of cytotoxicity observed on the BHK21 cells was not significant.

### *Winn neutralization assay*

To obtain further evidence in another system that the *in vitro* microcytotoxicity assay was reflecting the ability of the effector cells to destroy the target cell in

TABLE I.—*Cell Mediated Cytotoxicity of Progressor Peripheral Blood Lymphocytes on H50 and BHK21 Tumour Cells and Normal Hamster Fibroblasts*

| Expt | Target cells | E:T ratio | Effector source | Target cells remaining (mean<s.e.) | % reduction | *P* |
|---|---|---|---|---|---|---|
| 1 | H50 | 200 : 1 | Normal | 1285·8±73·9 | 28·4 | <0·0025 |
| | | | progressor* | 919·6±28·8 | | |
| | Normal fibroblasts | | Normal | 131·8±22·5 | −19·7 | NS |
| | | | progressor* | 157·7±18·6 | | |
| 2 | H50 | 150 : 1 | Normal | 464·3±22·8 | 84·0 | <0·0005 |
| | | | progressor† | 74·0±24·8 | | |
| | BHK21 | | Normal | 1799·4±139·3 | −2·4 | NS |
| | | | progressor† | 1843·4±174·3 | | |

* Progressively growing tumour derived from SV40 cells maintained in tissue culture.
† Progressively growing tumour derived from SV40 cells passaged *in vivo*.

TABLE II.—*Cell Mediated Cytotoxicity of Immune Peripheral Blood Lymphocytes of H50, BHK21 Tumour Cells and Normal Hamster Fibroblasts*

| Expt | Target cells | E:T ratio | Effector source | Target cells remaining (mean±s.e.) | % reduction | *P* |
|---|---|---|---|---|---|---|
| 1 | H50 | 400 : 1 | Normal | 510·6±41·1 | 87·7 | 0·0005 |
| | | | Immune | 63·0±13·1 | | |
| | | 200 : 1 | Normal | 1030·8±21·6 | 63·8 | 0·0005 |
| | | | Immune | 373·5±30·7 | | |
| | Normal fibroblasts | 400 : 1 | Normal | 676·6±71·4 | −25·9 | NS |
| | | | Immune | 851·8±36·5 | | |
| | | 200 : 1 | Normal | 992·5±36·0 | −2·2 | NS |
| | | | Immune | 1014·6±25·0 | | |
| 2 | H50 | 500 : 1 | Normal | 417·0±14·6 | 76·3 | 0·0005 |
| | | | Immune | 98·8±12·7 | | |
| | | 250 : 1 | Normal | 562·4±42·2 | 29·1 | 0·0005 |
| | | | Immune | 398·6±26·4 | | |
| | BHK21 | 250 : 1 | Normal | 525·5±64·5 | 12·6 | NS |
| | | | Immune | 459·1±53·9 | | |
| | | 125 : 1 | Normal | 895·8±65·8 | 10·6 | NS |
| | | | Immune | 746·4±51·9 | | |

TABLE III.—*Effect of Immune and Progressor Peripheral Blood Lymphocytes (PBL) On the Appearance of Tumours* in Vivo *in the Winn Assay*

| Expt | Cells injected | % of cheek pouches with tumours (weeks) 1 | 2 | 3 | 4 | 5 |
|---|---|---|---|---|---|---|
| 1 | Group A (4)* | | | | | |
| | $10^4$ H50 | 0 | 75 | 100 | 10 | 100 |
| | Group B (8) | | | | | |
| | $10^4$ H50+$10^6$ Normal PBL | 50 | 75 | 100 | 100 | 100 |
| | Group C (8) | | | | | |
| | $10^4$ H50+$10^6$ Immune PBL | 0 | 0 | 12·5 | 12·5 | 12·5 |
| 2 | Group D (8) | | | | | |
| | $10^4$ H50 | 0 | 0 | 50 | 60 | 75 |
| | Group E (6) | | | | | |
| | $10^4$ H50+$10^6$ Normal PBL | 0 | 33 | 50 | 60 | 75 |
| | Group F (10) | | | | | |
| | $10^4$ H50+$10^6$ Progressor PBL | 0 | 20 | 50 | 100 | 100 |

* Figures in parentheses indicate number of cheek pouches injected; odd percentages are due to the fact that 3 animals died during the course of the experiment for unexplained reasons.

question, we performed experiments using the tumour cell neutralization assay described by Winn (1961). In these experiments, normal, immune and progressor peripheral blood lymphocytes, isolated in the same manner as for the microcytotoxicity assay, were incubated with H50 tumour cells at an effector to target cell ratio of 100 : 1. These mixtures were then injected into the cheek pouch of normal hamsters which were examined weekly for the development of tumours. The results in Table III indicate a marked inhibition of the growth of H50 target cells pre-incubated with immune peripheral blood lymphocytes. This observation is in contrast to the failure of inhibition when the tumour cells were pre-incubated with progressor peripheral blood lymphocytes.

*Effect of progressor and post-excision regrowth serum on lymphocyte cytotoxicity*

Based on the general observation that serum blocking factors can be detected *in vitro* about the time a tumour becomes palpable and thereafter (Hellström and Hellström, 1974), we initially screened progressor serum for blocking activity. The results in Table IV, experiments 1 and 2, indicate that no abrogation of *in vitro* immune lymphocytotoxicity was obtained with serum from hamsters whose palpable tumour was less than 3 cm in the largest diameter (progressor-middle) and from hamsters whose palpable tumour was greater than 3 cm in size in the largest diameter (progressor-late).

As it may not be possible to detect serum neutralizing activity by the addition of the test serum to the target cells, progressor serum was incubated with the effector cells and then the effector cells were added to the target cells. Again, no abrogation of immune lymphocyte cytotoxicity was observed ( Table IV, experiment 3). These results directly contrast with the observations made with post-excision regrowth serum. This serum reduced the cytotoxic potential of immune lymphocytes from 62·4% to 23·6% in comparison with normal hamster serum (Table IV, experiment 4).

*Concomitant tumour immunity*

Experiments of concomitant tumour immunity (Bashford *et al.*, 1908) were performed as other studies reported that the presence of concomitant tumour immunity correlated with the absence of blocking factors (Deekers *et al.*, 1973; Sjögren and Bansal, 1971). $10^4$ H50 cells were injected intradermally in the intrascapular area of 32 hamsters and on the following 4 consecutive weeks groups of

TABLE IV.—*The Effect of Treating Normal and Immune Peripheral Blood Lymphocytes with Progressor and Post-excision Regrowth Serum on Their Cytotoxicity for Plated H50 Cells*

| Expt | Serum Donor | Target cells normal lymphocytes | Remaining± s.e. immune lymphocytes | % Reduction | % Blocking | Arming | % Potentiation | *P* |
|---|---|---|---|---|---|---|---|---|
| 1 | Normal | 2366·6±74·7 | 1303·6±97·9 | 44·9 | 6·2 | – | – | NS |
| | Progressor (Middle) | 2450·9±61·9 | 1419·9±67·1 | 42·1 | 6·2 | – | – | |
| 2 | Normal | 1217·1±89·9 | 611·9±73·0 | 49·7 | – | – | 0·4 | NS |
| | Progressor (Late) | 1339·3±72·7 | 670·5±61·0 | 49·9 | – | – | | |
| 3 | Normal | 728·5±25·2 | 269·6±25·2 | 62·9 | – | | | |
| | *Progressor (Late) | 681·5±42·4 | 240·1±17·5 | 64·7 | – | 6·5 | 2·9 | NS |
| 4 | Normal | 808·4±57·8 | 304·1±21·4 | 62·4 | | – | – | <0·0005 |
| | Post-excision Regrowth | 729·0±39·5 | 556·8±62·7 | 23·6 | 6·2 | – | – | <0·0005 |

* Neutralization experiment.

8 hamsters were challenged together with control hamsters with $10^3$ H50 cells subcutaneously in the groin. The results shown in Table V indicate that hamsters bearing a primary H50 tumour (palpable–2·0 cm size) showed a heightened resistance and not enhanced growth of a secondary challenge compared with normal controls.

*Tumour growth enhancement* in vivo

The ability of progressor serum to enhance tumour growth *in vivo* was also evaluated. Hamsters received an intradermal injection of H50 cells, followed by an intraperitoneal injection of one of the following sera: early; middle; late progressor or normal hamster serum. Weekly measurements of tumour size and incidence were recorded. The results (Table VI) do not give any indication of enhanced tumour growth or a large difference in tumour incidence when a comparison is made between the different progressor sera and normal hamster serum.

*Effect of progressor (early), immune and post-excision serum on lymphocyte cytotoxicity*

Classically, tumour immunity has been demonstrated in models where the animal was immunized with virus or virus transformed cells, or after complete excision of a progressively growing tumour. We wished to determine if immune and post-excision serum had an effect on tumour cell growth *in vitro*. The results shown in Table VII, experiments 2 and 3 indicate that immune (virus and transplantable) and post-excision sera potentiated the cytotoxic effect of immune effector cells and were also capable of arming normal effector cells. This arming and potentiating effect was also present in progressor serum (early) before the formation of a palpable tumour (Table VII, experiment 1).

*Mixed haemadsorption test*

The mixed haemadsorption antibody assay was used to determine whether

TABLE V.—*Concomitant Tumour Immunity*

| | Time of secondary challenge | Size of primary tumour* | Incidence of secondary tumours in weeks after primary challenge | | | | | | |
|---|---|---|---|---|---|---|---|---|---|
| | | | 1 | 2 | 3 | 4 | 5 | 6 | 7 |
| Tumour bearers | 1 Week | Palpable | – | – | 0/8 | 0/8 | 0/7 | 0/7 | 0/4 |
| Controls | | | – | – | 1/6 | 2/6 | 4/6 | 4/6 | 4/6 |
| Tumour bearers | 2 Weeks | 0·5 cm | – | – | – | 0/8 | 0/8 | 0/7 | 0/3 |
| Controls | | | – | – | – | 0/6 | 3/8 | 4/6 | 4/6 |
| Tumour bearers | 3 Weeks | 1·0 cm | – | – | – | – | – | 1/5 | 1/4 |
| Controls | | | – | – | – | – | – | 5/6 | 6/6 |
| Tumour bearers | 4 Weeks | 2·0 cm | – | – | – | – | – | 0/5 | 0/5 |
| Controls | | | – | – | – | – | – | 0/6 | 2/6 |

* Measurements refer to greatest single diameter

TABLE VI.—*Evaluation of Progressors Serum's* in Vivo *Enhancing Ability*

| | Weeks | | | |
|---|---|---|---|---|
| | 1 | 2 | 3 | 4 |
| $10^3$ Cell dose | | | | |
| Normal hamster serum | 2/6 | 3/6 | 4/6 | 3/5 |
| Early progressor serum | 0/4 | 2/4 | 2/4 | 2/4 |
| Middle progressor serum | 0/4 | 1/4 | 3/4 | 3/3 |
| Late progressor serum | 0/4 | 1/4 | 2/4 | 3/4 |
| $10^4$ Cell dose | | | | |
| Normal hamster serum | 3/6 | 4/6 | 4/6 | 2/3 |
| Early progressor serum | 1/4 | 3/4 | 3/4 | 3/4 |
| Middle progressor serum | 2/4 | 4/4 | 4/4 | 3/3 |
| Late progressor serum | 3/4 | 4/4 | 4/4 | 3/3 |

TABLE VII.—*The Effect of Treating Normal and Immune Peripheral Blood Lymphocytes with Progressor (Early), Post-excision and Immune Serum on Their Cytotoxicity for Plated H50 Cells*

| Expt | Serum donor | Target cells normal lymphocytes | Remaining ± s.e. immune lymphocytes | % Cytotoxicity | % Blocking | % Arming | % Potentiation | *P* |
|---|---|---|---|---|---|---|---|---|
| 1 | Normal | 808·4 ± 57·8 | 304·1 ± 21·4 | 62·4 | – | | | |
| | Progressor (early) | 551·6 ± 23·9 | 161·0 ± 20·2 | 70·8 | – | 31·8 | 11·8 | <0·0005 |
| 2 | Normal | 591·9 ± 46·3 | 191·3 ± 17·1 | 67·7 | – | 15·5 | 39·1 | <0·0005 |
| | Post-excision | 500·1 ± 51·8 | 28·6 ± 13·3 | 94·2 | | | | |
| 3 | Normal | 976·8 ± 53·1 | 627·3 ± 44·7 | 35·8 | | | | |
| | Immune virus | 724·3 ± 42·2 | 172·0 ± 11·9 | 76·2 | – | 25·8 | 112·8 | <0·0005 |
| | Immune transplantable | 646·4 ± 87·9 | 245·5 ± 13·4 | 62·0 | – | 33·8 | 73·8 | <0·0005 |

TABLE VIII.—*Mixed Haemadsorption Test*

| Serum | Cell line | Titration 1/2–1/64 | Titration 1/10–1/10,000 |
|---|---|---|---|
| Progressor early | H50,SV3T3 | – | ND |
| Progressor middle | H50,SV3T3 | – | – |
| Progressor late | H50,SV3T3 | – | – |
| Post-excision | H50,SV3T3 | – | – |
| Post-excision (regrowth) | H50,SV3T3 | – | ND |
| Immune transplantable | H50,SV3T3 | – | – |
| Immune virus | H50,SV3T3 | – | – |
| Hamster anti-mouse tissue | L cells | + | + |

antibodies to the tumour cell surface could be detected in the various sera under study. Each serum was tested on a minimum of 2–4 SV40 transformed cell lines. The results shown in Table VIII indicate that progressor (early, middle, late) post-excision (clean and regrowth) and immune (virus, transplantable) sera constantly gave negative results. The sera were titrated both in a narrow and wide range to rule out any prozone effect. Enzymatic treatment (trypsin, neuraminidase) of the target cells did not alter these results, thus ruling out potential masked antigens as a reason of unreactivity. In contrast, an antiserum raised in hamsters to normal mouse tissue could be titrated to a dilution of $10^{-4}$ against normal mouse fibroblasts.

### DISCUSSION

This study was initiated to see if blocking factors were present in the serum of hamsters bearing a transplantable SV40 tumour. The study of blocking factors requires an *in vitro* assay of cell mediated immunity that parallels the *in vivo* response. Peripheral blood lymphocytes isolated from hamsters bearing 3 cm, SV40 tumours demonstrated specific *in vitro* lymphocytotoxicity but could not neutralize tumour growth in a Winn test. Similar observations have been made by Coggin *et al.* (1974) with SV40 autochthonous tumours and in other tumour–host systems (Howell, Dean and Law, 1975). The study of Howell *et al.* (1975) suggested that the inability to neutralize tumour growth was due to a lesion in the T cell system of the tumour bearing host. Thus, progressor peripheral blood lymphocytes isolated from hamsters bearing 3 cm tumours would not be an appropriate effector population to evaluate serum blocking factors even though these effector cells demonstrated specific cell mediated cytolysis in the microcytotoxicity assay. In contrast, the positive *in vitro* anti-tumour effect of immune peripheral blood lymphocytes demonstrated with the microcytotoxicity assay and Winn neutralization test correlated with the tumour rejection observed in the intact animal. Consequently, immune peripheral blood lymphocytes were chosen as the effector population to evaluate potential serum blocking factors in the hamster SV40 system.

This study observed two other effects of serum besides blocking of *in vitro* lymphocytotoxicity. Thus, besides blocking, each serum had no effect on the specific cell mediated anti-tumour cytotoxic response, or it potentiated the response. The pattern of serum response appears to correlate better with resistance to tumour growth than does the lymphocyte reactivity observed in the microcytotoxicity assay.

Contrary to numerous other animal tumour systems (Hellström *et al.*, 1974), serum blocking factors were not a common occurrence in association with progressive tumour growth in our model. In fact, the only situation where we found serum blocking factors was in those animals who experienced a recurrence of a previously excised tumour. It is important to stress that serum was tested for blocking activity, both at the target and effector cell level. In tests of concomitant tumour immunity enhanced growth *in vivo* was not seen in animals with progressively growing tumours, nor did serum taken from these animals cause enhanced tumour growth when passively transferred to normal hosts. Unfortunately, we have not been able to perform similar studies with sera demonstrating *in vitro* blocking activity (post-excision regrowth).

Serum taken from animals with progressively growing tumours neither blocked nor potentiated cell mediated cytotoxicity. The only exception to this was serum taken from animals shortly after inoculation with a clearly tumourigenic number of H50 cells, but before the clinical appearance of tumour. Such sera demonstrated characteristics similar to sera taken from animals rendered immune to tumour transplantation by various procedures. These immune sera augmented the cytotoxic reactivity of tumour immune lymphocytes *in vitro* (potentiation) (Lamon *et al.*, 1974) and also induced normal lymphocytes to specifically kill tumour cells (arming) (Basham and Currie, 1974).

The nature of the serum factors involved in these phenomena are not clear from the studies which we have performed to date. No evidence of antibody directed against the tumour cell surface was obtained using mixed haemadsorption tests. The sera alone were not cytotoxic and attempts to lyse the tumour cells with such sera in the presence of complement in classic humoral cytotoxicity assays were unsuccessful (unpublished observations).

There is a strong parallel between those situations in which we have been able to demonstrate an antibody dependent cellular cytotoxicity effect and those in which Ambrose, Anderson and Coggin (1971) demonstrated cytostatic antibody activity. Whether the antibody component of these two phenomena is identical or whether two different parameters of antitumour immunity are involved is not known and requires further study.

Of major importance we feel, is the fact that there appears to be some relevance of the antibody dependent cellular cytotoxicity reaction to the actual tumour host interaction which is not revealed by classic lymphocytotoxicity tests. Thus, progressive primary tumour growth may correlate with a lack of the antibody component of the antibody dependent cellular cytotoxicity response rather than with the presence of a serum blocking factor. A second area of importance is the relative rarity of blocking factors in our model as a feature of progressive tumour growth. Metastasis in this model is a rare and very late event, so that it is conceivable that at least in this system blocking factors may correlate more with the presence of recurrent or metastatic tumour rather than with progressive tumour growth alone.

Supported by a grant from the Medical Research Council of Canada. We wish to thank Mrs G. Vincze, Miss Debbie Liebow and Mrs G. Klein for technical assistance and Mrs Beth Meyer for secretarial support in preparation of this manuscript.

### REFERENCES

ALEXANDER, P. (1974) Escape from Immune Destruction by the Host Through Shedding of Surface Antigens: Is This a Characteristic Shared By Malignant and Embryonic Cells? *Cancer Res.*, **34**, 2077.

AMBROSE, K. R., ANDERSON, N. G. & COGGIN, J. H. JR (1971) Cytostatic Antibody and SV40 Tumour Immunity in Hamsters. *Nature, Lond*, **233**, 321.

ASHKENAZI, A. & MELNICK, J. L. (1963) Tumorogenicity of Simian Papovavirus SV40 and of Virus-transformed Cells. *J. natn. Cancer Inst.*, **30**, 1227.

BALDWIN, R. W., PRICE, M. R. & ROBINS, R. A. (1973) Inhibition of Hepatoma-Immune Lymphnode Cell Cytotoxicity by Tumour Bearer Serum and Solubilized Hepatoma Antigen. *Int. J. Cancer*, **11**, 527.

BARTH, R. F., ESPMARK, J. A. & FAGRAEUS, A. (1967) Histocompatability and Tumor Virus Antigens Identified on Cells Grown in Tissue Culture by Means of Mixed Hemadsorption Reaction. *J. Immun.*, **98**, 888.

BASHAM, C. & CURRIE, C. A. (1974) Development of Specific Cell-dependent Antibody During Growth of a Syngeneic Rat Sarcoma. *Br. J. Cancer*, **29**, 189.

BASHFORD, E. F., MURRAY, J. A., HAALAND, M. & BOWEN, W. H. (1908) General Results of Propagation of Malignant New Growths. Third Scientific Report of the Imperial Cancer Research Fund, **3**, 262.

BOYUM, A. (1968) Separation of Leucocytes from Blood and Bone Marrow. *Scand. J. clin. Lab. Invest.* **21**, Suppl. 97, 1.

BUTEL, J. S., TEVETHIA, S. S. & MELNICK, J. L. (1971) Oncogenicity and Cell Transformation by Papovavirus SV40: The Role of the Viral Genome. *Adv. Cancer Res.*, **15**, 1.

COGGIN, J. H. JR & ANDERSON, N. G. (1972) Phase-specific Autoantigens (Fetal) in Model Tumor Systems. In *Embryonic and Fetal Antigens in Cancer*. Ed. N. G. Anderson, J. H. Coggin Jr, E. Cole & J. W. Holleman. Springfield, Va.: United States Department of Commerce. Vol. **2**. p. 72.

COGGIN, J. H. JR, AMBROSE, K. R., DIERLMAN, P. J. & ANDERSON, N. G. (1974) Proposed Mechanisms by Which Autochthonous Neoplasms Escape Immune Rejection. *Cancer Res.*, **34**, 2092.

CURRIE, G. A. & BASHAM, C. (1972) Serum Mediated Inhibition of the Immunological Reactions of the Patient to His Own Tumour: A Possible Role for Circulating Antigen. *Br. J. Cancer*, **26**, 427.

CURRIE, G. A. & ALEXANDER, P. (1974) Spontaneous Shedding of Tumour Specific antigens by Viable Sarcoma Cells. Its Possible Role in Facilitating Metastatic Tumour Spread. *Br. J. Cancer*, **29**, 72.

DEEKERS, P. J., DAVIES, R. C., PARKER, G. A. & MANICK, J. A. (1973) The Effect of Tumor Size on Concomitant Tumor Immunity. *Cancer Res.*, **33**, 33.

DEICHMAN, G. I. & KLUCHAREVA, T. E. (1964) Immunological Determinants of Oncogenesis in Hamsters Infected with SV40 Virus. *Virology*, **24**, 131.

DELANDAZURI, M. O., KEDAR, E. & FAHEY, J. L. (1974). Antibody Dependant Cellular Cytotoxicity to a Syngeneic Gross Virus-induced Lymphoma. *J. natn. Cancer Inst.*, **52**, 147.

GIRARDI, A. J. (1966) Tumor Resistance and Tumor Enhancement with SV40 Virus-Induced Tumors. In *Germinal Centers in Immune Responses*. New York: Springer Verlag. p. 422.

HELLSTRÖM, K. E. & HELLSTRÖM, I. (1974) Lymphocyte-mediated Cytotoxicity and Blocking Serum Activity to Tumor Antigens. In *Advances in Immunology*. Eds. F. J. Dixon & H. G. Kunkel. New York: Academic Press, **18**, 209.

HELLSTRÖM, I., HELLSTRÖM, K. E. & WARNER, G. A. (1973) Increase of Lymphocyte Mediated Tumor Cell Destruction by Certain Patient Sera. *Int. J. Cancer*, **12**, 348.

HOWELL, S. B., DEAN, J. H. & LAW, L. W. (1975) Defects in Cell-mediated Immunity During Growth of a Syngeneic Simian Virus-induced Tumor. *Int. J. Cancer*, **15**, 152.

LAMON, E. W., ANDERSON, B., WIGZELL, H., FENYO, E. M. & KLEIN, E. (1974) The Immune Response to Primary Moloney Sarcoma Virus Tumors in BALB/c Mice. Cellular and Humoral Activity of Long Term Regressor. *Int. J. Cancer*, **13**, 91.

MACLENNAN, I. C. M. (1972) Antibody in the Induction and Inhibition of Lymphocyte Cytotoxicity. *Transplantn Rev.*, **13**, 67.

MACPHERSON, I. A. & STOKER, M. P. G. (1962) Polyome Transformation of Hamster Cell Clones —An Investigation of Genetic Factors Affecting Cell Competence. *Virology*, **16**, 147.

POLLACK, S., HEPPNER, G., BRAWN, R. J. & NELSON, K. (1972) Specific Killing of Tumor Cells *in vitro* in The Presence of Normal Lymphoid Cells and Sera From Hosts Immune to the Tumor Antigens. *Int. J. Cancer*, **9**, 316.

SJÖGREN, H. D. & BANSAL, S. C. (1971) Antigens. In *Virally Induced Tumors*. Ed. B Amos. *Prog. Immunol*, p. 921.

SJÖGREN, H. O., HELLSTRÖM, I., BANSAL, S. C. & HELLSTRÖM, K. E. (1971) Suggestive Evidence That the Blocking Antibodies of Tumor-bearing Individuals May be Antigen–Antibody Complexes. *Proc. natn. Acad. Sci.* U.S.A., **68**, 1372.

TAKASUGI, M. & KLEIN, E. (1970) A Microassay for Cell-Mediated Immunity. *Transplantation*, **9**, 219.

WINN, H. J. (1961) Immune Mechanisms in Homotransplantation: II. Quantitative Assay of the Immunologic Activity of Lymphoid Cells Stimulated by Tumor Homografts. *J. Immun.*, **86**, 228.

ZARLING, J. M. & TEVETHIA, S. S. (1973*a*) Transplantation Immunity to Simian Virus 40-Transformed Cells in Tumor-bearing Mice. I. Development of Cellular Immunity to Simian Virus 40 Tumor Specific Transplantation Antigens During Tumorogenesis by Transplanted Cells. *J. natn. Cancer Inst.*, **50**, 137.

ZARLING, J. M. & TEVETHIA, S. S. (1973*b*) Transplantation Immunity to Simian Virus 40-Transformed Cells in Tumor-bearing Mice. II. Evidence for Macrophage Participation at the Effector Level of Tumor Cell Rejection. *J. natn. Cancer Inst.*, **50**, 149.